# Expanding registered reports

**DOI:** 10.1038/s41467-022-34107-w

**Published:** 2022-11-28

## Abstract

Since 2020 *Nature Communications* has been considering Registered Reports for publication in the areas of cognitive neuroscience, human behaviour and psychology, and epidemiology. We are excited to announce the publication of our first Registered Report. With this milestone, we also want to open the format to all other areas of research.

The launch of Registered Reports in 2020, and now our first publication, represent an important step in our commitment to encouraging transparent research and publishing practices that avoid wasting resources by ensuring upfront that the methods used are as strong as they can be to address the research question. Our first registered report, by Magda Dubois and Tobias Hauser, entitled “Value-free random exploration is linked to impulsivity,” shows that impulsivity is associated with an increased usage of a cognitively cheap, and sometimes sub-optimal, exploration strategy.

In Registered Reports, the study proposal is peer reviewed and approved before the sampling and experimental design is settled and the research is undertaken. Input from reviewers helps authors in shaping the proposal, and the results of the study are published regardless of the outcome, provided the study is completed according to the accepted proposal. The goal of this format is to reduce the extent to which authors, reviewers, and editors evaluate research based on its outcome, and for all stakeholders to focus instead on the importance of the research question being tested and on the extent to which the proposed methods can provide a clear answer to that question. By requiring authors to publicly commit to a study plan, Registered Reports also help to reduce opportunities to engage in questionable research practices, such as p-hacking and HARKING, which contribute to biases towards publishing positive results. Indeed, recent work provides evidence that Registered Reports are of higher quality methodologically and analytically than non-registered reports.

We have received more than 50 submissions so far and most of these were in the areas of human behaviour and psychology. To further solidify our commitment to this format, we are now opening it up to all areas of research, across the full breadth of science published at *Nature Communications*. We are also particularly excited to participate in a Registered Reports Funding Partnership pilot with Cancer research UK alongside 12 other journals. The initiative aims to streamline the Registered Report process and to provide an opportunity to gain expert feedback on the study plan as soon as funding is warranted and before collecting the data as well as reassurance of a publication at the end. The format is particularly well suited for hypothesis driven research, which is why Cancer Research UK chose their ‘Early Detection and Diagnosis’ and ‘Prevention and Population Research’ Project Awards to participate in this initial pilot in light of the studies they usually fund.

In addition to these examples, we believe that there are other types of studies that would benefit greatly from the format. Following in the footsteps of Nature Methods, we are strongly encouraging researchers undertaking comparative analyses measuring and ranking the performance of established tools or methods to submit a Registered Report. It is our hope that large-scale analyses of comparable methods will benefit greatly from peer-review of their study design before they begin to help ensure that they can answer the question asked and better benefit the community.

For many research communities, this article format will appear new and perhaps daunting at first. We have detailed guidelines to help both researchers and reviewers during the process and we are available to answer questions you may have. Authors and one reviewer of our first Registered Report are sharing their experience in a Q&A published alongside this editorial. Although evaluation of the Stage 1 study design can require multiple interactions with reviewers to ensure its robustness. Once the study is approved, publication is guaranteed, provided that the study plan was followed, pre-specified quality checks are passed and the interpretation of the results is appropriate and defensible. After data are collected, analysed, and reported, the evaluation of the Stage 2 full report is often relatively straightforward. We encourage researchers from all areas to consider whether the format will be suitable for their project when they are engaging in hypothesis testing and confirmatory research, or when designing a resource intensive long-term experiment or large-scale observational study. In a second Q&A, Shinichi Nakagawa and Rose O’Dea discuss how the Registered Reports format would be suitable and useful for many projects in ecology and evolution.

“We encourage researchers from all areas to consider whether the format will be suitable for their project when they are engaging in hypothesis testing and confirmatory research, or when designing a resource intensive long-term experiment or large-scale observational study.”

Even when it is not possible to submit a Registered Report, for example if a project has already started or it is purely exploratory, we would recommend authors to consider pre-registering a research plan and making it available through a read-only public repository. This is becoming wide-spread practice in psychology and social sciences studies. However, regardless of your field of research, pre-registering your study plan can improve the transparency and discoverability of your research.

Looking forward, the editors at *Nature Communications* are excited about the publication of more Registered Reports which we will collate as they are published in a dedicated Collection. We are also interested in hearing from people who would like to get involved in reviewing these papers; if you have prior experience publishing or reviewing Registered Reports, we would like to hear about your experience. These comments would be helpful to us in considering how we might revise our author and reviewer guidelines to further improve the quality of the review process.

